# A Promising Candidate to Reliably Index Attentional Bias Toward Alcohol Cues–An Adapted Odd-One-Out Visual Search Task

**DOI:** 10.3389/fpsyg.2021.630461

**Published:** 2021-02-10

**Authors:** Janika Heitmann, Nienke C. Jonker, Peter J. de Jong

**Affiliations:** ^1^Verslavingszorg Noord Nederland, Groningen, Netherlands; ^2^Department of Clinical Psychology and Experimental Psychopathology, University of Groningen, Groningen, Netherlands

**Keywords:** attentional bias, alcohol use, addiction, reliability, internal consistency, visual search

## Abstract

Attentional bias (AB) has been suggested to contribute to the persistence of substance use behavior. However, the empirical evidence for its proposed role in addiction is inconsistent. This might be due to the inability of commonly used measures to differentiate between attentional engagement and attentional disengagement. Attesting to the importance of differentiating between both components of AB, a recent study using the odd-one-out task (OOOT) showed that substance use was differentially related to engagement and disengagement bias. However, the AB measures derived from the OOOT showed insufficient reliability to be used as a solid measure of individual differences. Therefore, the current study aimed to improve the reliability of the AB measures derived from the OOOT by using more distinct contrast stimuli, adding practice trials, increasing the number of trials, and by having participants perform the task in an alcohol-relevant context. We contrasted the original OOOT with the adapted OOOT (i.e., OOOT-adapt) and assessed AB in low- and high-drinking individuals. Participants were 245 undergraduate students who typically tend to drink either low or high amounts of alcohol. In one condition, AB was measured with the original OOOT in a typical laboratory context, whereas in the other condition, AB was measured with the OOOT-adapt in a bar (i.e., alcohol-relevant) context. The OOOT-adapt showed superior internal consistency, especially for the high-drinking group. Further, specifically the OOOT-adapt differentiated between low- and high-drinking participants showing that high drinkers engaged faster with alcohol cues than did low drinkers. Thus, the OOOT-adapt was found to be a promising candidate to reliably index AB in the context of alcohol use. The OOOT-adapt further showed superior criterion validity as it could differentiate between low- and high-drinking individuals, thereby adding to the evidence that AB might be involved in substance use behavior.

## Introduction

Dual process models of addiction attribute an important role to automatic processes when explaining the development and persistence of addiction (Wiers et al., 2007; Stacy and Wiers, 2010). One of these processes is biased selective attention, also referred to as attentional bias (AB). AB can be expressed by a relatively strong tendency to direct attention to substance-relevant cues in the environment (i.e., engagement bias) and/or by a difficulty to redirect attention away from these cues (i.e., disengagement bias; Posner, 1980; Posner and Petersen, 1990). Although, in general, the contributing role of AB to the persistence of addictive behavior has been extensively studied throughout the past 15 years, only little is known about the specific role of engagement and disengagement bias. Directly distinguishing between engagement and disengagement bias might not only help to improve the general understanding of the disorder, but might also deliver knowledge that can be used to improve treatment (see, for example, Rinck et al., 2005; Hollitt et al., 2010).

One important reason for the limited knowledge about the role of engagement and disengagement bias relates to the fact that most measures of AB, such as the visual probe task (MacLeod et al., 1986), the addiction Stroop task (Cox et al., 2006), the flicker-induced change blindness task (Jones et al., 2002), or more recently developed tasks (e.g., Pennington et al., 2020), are not configured to differentiate between these two underlying processes of attention (Field and Cox, 2008; Grafton and MacLeod, 2014). That is, these assessment tasks deliver one overall index for AB. There are studies using for example the visual probe task, which aimed to disentangle engagement and disengagement bias by the use of different stimulus presentation durations (i.e., brief durations to index engagement bias and longer durations to index disengagement bias; e.g., Bradley et al., 2003; Field et al., 2006; Noël et al., 2006). Although this approach provided relevant information about initial and maintained attention, it has been pointed out that the use of different stimulus presentation intervals in the visual probe task does not allow disentangling engagement and disengagement processes (Grafton and MacLeod, 2014). One task that is configured to deliver separate indices for engagement bias and disengagement bias is the so-called odd-one-out task (OOOT; Hansen and Hansen, 1988; Rinck et al., 2005), which has been successfully used in previous research including studies on anxiety (De Voogd et al., 2017), sexual pain disorders (Melles et al., 2016), and eating behavior (Jonker et al., 2019). In the OOOT, participants are presented with an array of multiple stimuli identifying whether these stimuli are from the same category of images or whether one stimulus is defiant from the others (i.e., an odd-one-out). The task includes trials in which (1) all images are either disorder-relevant or disorder-irrelevant; (2) a disorder-relevant image is presented among disorder-irrelevant distractors; (3) a disorder-irrelevant image is presented among disorder-relevant distractors; and (4) a disorder-irrelevant image is presented among disorder-irrelevant distractors. The last trial type allows calculating a baseline of how long it generally takes to identify an odd-one-out among distractors allowing to calculate separate indices for engagement and disengagement bias by contrasting the reaction time of this neutral trial type with the other two trial types including disorder-relevant images. That is, engagement bias is expressed by the difference between trials in which a disorder-relevant image is presented among disorder-irrelevant distractors and the neutral trial type, whereas disengagement bias is expressed by the difference between trials in which a disorder-irrelevant image is presented among disorder-relevant distractors and the neutral trial type.

First indication that the OOOT also seems useful in examining engagement and disengagement bias in the context of alcohol use comes from a previous study from our laboratory in which, in a student sample, it was found that the disengagement index of the OOOT, but not the engagement index, was related to alcohol consumption, meaning that consuming higher amounts of alcohol was related with more difficulty to disengage attention from alcohol cues (Heitmann et al., 2020). However, the robustness of these findings may be questioned as the results indicated unacceptably low internal consistency of the AB indices. Yet, especially when being used as a measure of individual differences, it is critical that indices of AB show adequate reliability (e.g., McNally, 2019), and the commonly found low reliability of popular AB measures has been highlighted as a major threat for progress within this field of research (Rodebaugh et al., 2016). Therefore, the current study was designed to take up the challenge to modify the OOOT in a way to reach an acceptable level of reliability.

There are several aspects that might explain the low internal consistency of the AB indices as calculated from the original OOOT in the previous study (Heitmann et al., 2020). That is why we made several improvements to the design in the current study. First, the OOOT was improved by using more distinct contrast categories. That is, in the previous study, the neutral contrast categories (i.e., soft drinks and flowerpots) of the OOOT might have been insufficiently distinct from the target stimuli (i.e., alcoholic drinks), as we found that participants tend to make a substantial number of mistakes when following the task instruction to indicate whether a trial included an odd-one-out. Other studies, using more distinct contrast categories that were visually as well as content-wise less similar to the target category, have reported lower error rates and better internal consistency (e.g., Jonker et al., 2019). Second, the OOOT was further improved by adding practice trials including feedback, as well as adding more trials of trial types that are crucial to compute the AB indices (i.e., trial types including an odd-one-out). This seemed relevant as, in the previous study, only a limited number of trials of the OOOT were available to compute the AB indices (i.e., due to its configuration and high error rate), and a sufficient number of trials are necessary to reliably measure AB (Ataya et al., 2012). Third, the current study assessed AB in an alcohol-relevant context, as it has been shown that contextual factors might influence the stability of AB indices (Field et al., 2014; Christiansen et al., 2015). Fourth, AB was assessed in two groups, namely, low-drinking participants (i.e., low-alcohol group; 1–7 standard units a week) and high-drinking participants (i.e., high alcohol group; at least 14 standard units of alcohol a week). Thereby, we could test whether AB measures are more stable when assessing individuals for which alcohol cues are relatively salient/motivationally relevant—more likely individuals who drink higher amounts of alcohol (Field and Christiansen, 2012). Given that the previous study included a student sample in which the amount of used alcohol varied from little to high, the task might not have measured the processes of interest as, at least for the participants drinking little alcohol, the alcohol cues might have been less motivationally relevant (i.e., no AB for alcohol cues; Heitmann et al., 2020).

To follow up on the previous study and to investigate whether the internal consistency could be improved by using more distinct non-alcohol contrast stimulus categories, adding practice trials and increasing the number of trials, by having participants perform the task in a relevant context, and by assessing AB in low- and high-drinking individuals, we compared the internal consistency of this new and improved task, called the OOOT-adapt, with the original OOOT. First, we hypothesized that the OOOT-adapt would show better internal consistency than the OOOT, which would be especially evident in the high-alcohol group. Second, we expected students in the high-alcohol group to show stronger AB to alcohol cues than students in the low-alcohol group. And finally, we hypothesized that if indeed internal consistency of the OOOT-adapt is superior compared to the internal consistency of the OOOT, the difference between the low- and high-alcohol group would be more pronounced when AB was measured with the OOOT-adapt.

## Materials and Methods

This study was preregistered with OSF and can be accessed via the following link^1^.

### Participants

Participants signed up for the study via an online participant platform. There were two advertisements on this platform, one recruiting individuals who drink low amounts of alcohol (low-alcohol group; 1–7 units per week) and one recruiting individuals who drink high amounts of alcohol (high-alcohol group; 14 units or more per week). Based on power analyses on the main analyses, a medium effect size of 0.6, power of 95%, and an α level of 0.05, we aimed for a sample size of 122 participants in each group. This was in line with previous studies showing a medium effect size when differentiating between groups using an AB task (e.g., Grafton and MacLeod, 2014). Eventually, 245 undergraduate students (46% male, mean_*age*_ = 20.3, SD_*age*_ = 2.08) from the psychology bachelor program of the University of Groningen participated in the study.

### Materials

#### Alcohol Use and Craving

The Measurements in Addiction for Triage and Evaluation Questionnaire (MATE-Q; Schippers and Broekman, 2014) was used to assess the quantity and frequency of alcohol use in the past 30 days, as well as craving for alcohol in the past 7 days. Quantity of use was indexed by summing the amount of standard glasses of alcohol consumed on a typical Monday, Tuesday, etc. This sum score was then multiplied by four to represent the amount of alcohol consumed in a typical month. Frequency of use was indexed by the question: “How often in the last 30 days have you used alcohol?” Alcohol craving was indexed by the Obsessive–Compulsive Drinking Scale (OCDS5) of the MATE-Q. The OCDS5 consists of five items measuring the desire for alcohol in the past 7 days, answered on a 5-point Likert scale. Alcohol craving was calculated by the sum of all items. Internal consistency of the OCDS5 was poor (Cronbach α of 0.51). This seemed to be related to item 4 of this questionnaire (i.e., “How much of an effort do you make to resist these thoughts or try to disregard or turn your attention away from these thoughts as they enter your mind?”). In line with our previous study (Heitmann et al., 2020), this item was therefore excluded, resulting in an acceptable internal consistency of the sum score of the remaining four items (Cronbach α of 0.70).

#### Alcohol Use Problems

Alcohol use–related problems were indexed with the shorted version of the Rutgers Alcohol Problem Index (RAPI-18; White and Labouvie, 1989). Participants had to indicate how often they experienced the 18 described situations in the past, using a 5-point Likert scale ranging from “never” (1) to “very often” (5). Per participant, a sum of scores was calculated. Internal consistency of the RAPI-18 was good (Cronbach α of 0.86).

#### Attentional Bias to Alcohol

**FIGURE 1:**
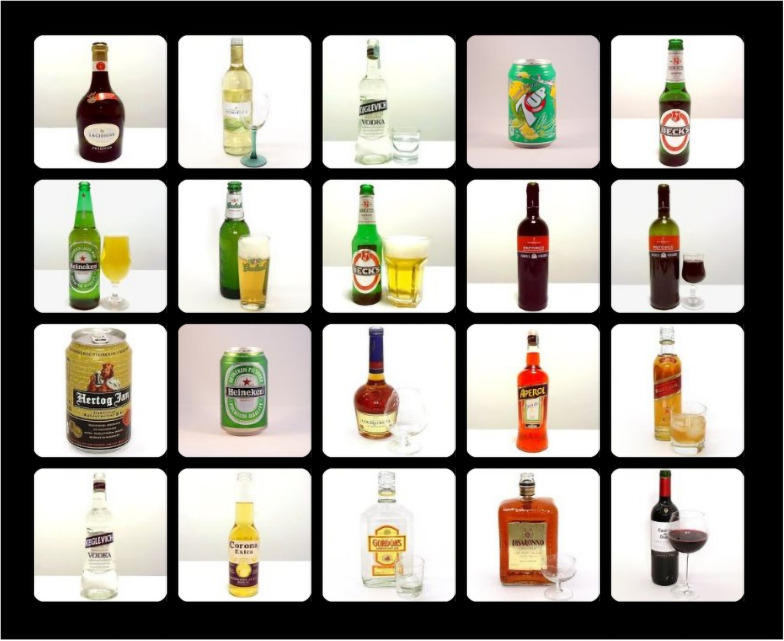
Example trial of the OOOT—an alcohol distractors trial.

Attentional bias to alcohol cues was measured with the original OOOT, as used in Heitmann et al. (2020), or the adapted version of the OOOT (OOOT-adapt). During the original OOOT, participants focused their attention on a red fixation cross in the center of the screen for 500 ms after which they had to indicate as quickly and correctly as possible whether there was an odd-one-out image within a 5 × 4 image matrix (500 × 500 pixels) by pressing the “0” (no odd-one-out) or “1” (yes, odd-one-out present) button on the keyboard. The task consisted of 54 trials with an odd-one-out and 18 trials without an odd-one-out (72 trials in total). The task was divided into three blocks of 24 trials. There were no practice trials in this task. The task consisted of three types of odd-one-out-present trials: alcohol target trials, with an alcohol odd-one-out and neutral (soft drinks or flower pots) distractors; alcohol distractors trial, with alcohol distractors and a soft drink or flower pot odd-one-out; neutral target in neutral distractors trial, with a soft drink odd-one-out in flower pot distractors; or a flower pot odd-one-out in soft drinks distractors. The three trial types without an odd-one-out consisted of either 20 alcohol images, 20 soft drink images, or 20 flower pot images. All trial types were randomly presented, and odd-one-out images randomly appeared over the possible positions, with the exception of directly above or below the fixation cross. Attentional engagement and attentional disengagement were inferred from trials in which an odd-one-out was present. Engagement bias was calculated by subtracting the mean response latency of alcohol target trials from the mean response latency of neutral target in neutral distractors trials. More attentional engagement with alcohol cues is then reflected in higher (more positive) scores. Disengagement bias was calculated by subtracting the mean response latency of neutral target in neutral distractors trials from the mean response latency of alcohol distractors trials. More difficulty to disengage attention from alcohol cues is reflected in higher positive scores. See Figure 1 for an example of a trial from the OOOT.

**FIGURE 2:**
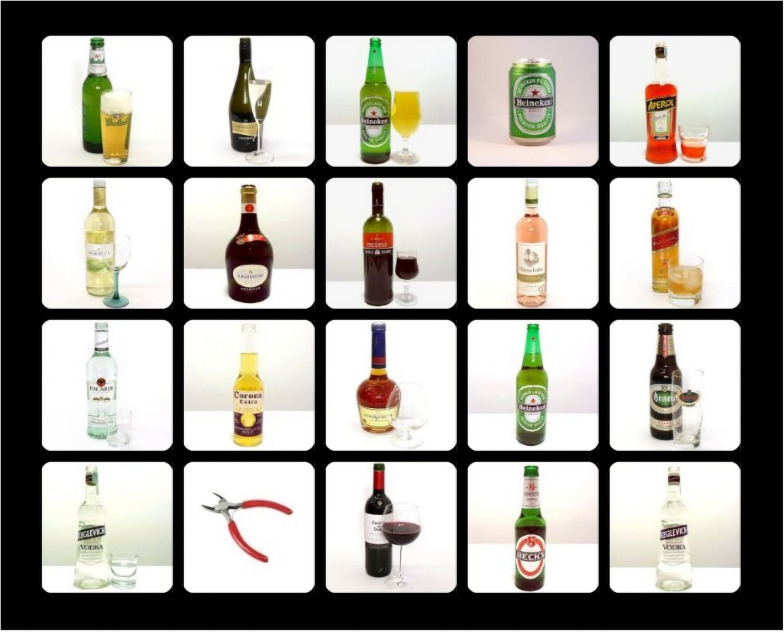
Example trial of the OOOT-adapt—an alcohol distractors trial.

The OOOT-adapt differed from the original OOOT in the following aspects: (1) the OOOT-adapt included at least 12 practice trials during which participants received feedback. If necessary, the number of practice trials was increased by one until a participant correctly responded to at least nine trials; (2) the OOOT-adapt consisted of 162 trials with 126 odd-one-out trials and 36 trials without an odd-one-out, divided into three blocks of 54 trials each; (3) the neutral distractors were images of office supplies and flowers instead of soft drinks and flower pots (see Heitmann et al., 2020). See Figure 2 for an example of a trial of the OOOT-adapt.

### Procedure

This study was approved by the ethical committee of the psychology department of the University of Groningen (PSY-1819-S-0081 and PSY-1819-S-0082). From the low-alcohol group and the high-alcohol group, half of the participants were assigned to the original OOOT and half to the OOOT-adapt. Participants were not aware that there were two different versions of the task (i.e., two conditions). On top of the adaptations to the task (see materials), also the location in which the OOOT-adapt was performed was different from the location in which the OOOT was performed. That is, the original version of the task was performed in a laboratory where assessment took place throughout the whole day similar to the study of Heitmann et al. (2020), whereas the adapted version of the task was performed in an alcohol-relevant context after 3 PM in the afternoon, i.e., a bar. To ensure that the location would not reveal the two different versions of the task, or bias the participants who would sign up, information about the location was given only 12 h prior to participants’ appointment. At that time, the online participant platform no longer accepted switching time slot.

The procedures of both versions of the study were similar. On entry to the laboratory or bar, participants signed informed consent. Then they indicated their gender and age. They also reported on their state alcohol craving by answering how much they currently craved alcohol on a 7-point Likert scale ranging from “no craving” (1) to “a lot of craving” (7). Hereafter participants completed the OOOT or OOOT-adapt, followed by the MATE-Q and the RAPI-18. Given the difference in the number of trials between the OOOT and the OOOT-adapt, participants completing the OOOT needed approximately 20 min to complete the study, whereas participants completing the OOOT-adapt needed approximately 30 min. All participants received course credits in return for their participation. For the first 16 participants, the RAPI-18 was erroneously not included in the study.

### Analyses Plan

#### Data Reduction of OOOT and OOOT-Adapt

Data reduction was performed separately for both conditions (OOOT and OOOT-adapt) and both groups (low- and high-alcohol groups). Participants who fell more than three SDs below the mean accuracy of their condition and group were excluded. As a next step, trials with incorrect responses were deleted. Further, trials in which participants respond faster than 200 ms (i.e., expected anticipation errors) or fall more than three SDs below or above their mean response latency of that trial type were excluded.

#### Hypothesis 1

To examine whether the OOOT-adapt showed better internal consistency than the original version of the OOOT, internal consistency of the OOOT and the OOOT-adapt was calculated, per group (i.e., low- and high-alcohol groups), in two different ways: (1) a split-half Spearman–Brown coefficient was calculated from the outcomes of the tasks based on the trials of the first half and the second half of the tasks; and (2) a second method in which Spearman–Brown coefficients were calculated from outcome measures based on half of the trials where we distributed the trials alternately to one of two subsets. The first trial of one particular trial type was randomly allocated to either of the two subsets. Internal consistency was calculated for the engagement and disengagement indices of both tasks. The Fisher *Z* test was used to statistically compare the internal consistency coefficients of the engagement and disengagement indices as calculated from the OOOT and the OOOT-adapt.

#### Hypothesis 2

To examine whether students of the high-alcohol group showed a stronger AB to alcohol cues than students in the low-alcohol group, we performed one-tailed independent *t* tests comparing students drinking low amounts of alcohol, with students drinking high amounts of alcohol. We examined group differences for the OOOT and OOOT-adapt separately. Per condition, two independent *t* tests were performed, one on attentional engagement and one on attentional disengagement. Given multiple comparisons per group (engagement and disengagement bias), for the one-tailed independent *t* tests, we used an adjusted α of 0.025, to reduce the likelihood of incorrectly rejecting the null hypothesis (i.e., making a Type I error).

To increase confidence in our results delivered by the *t* tests following the frequentist approach, we also reported results following the Bayesian approach. Therefore, Bayesian independent-samples *t* tests with Cauchy priors were calculated, which are set at the recommended default *r* = 0.707. BF_10_, which quantifies the evidence for the alternative hypotheses over the null hypotheses, was reported. A Bayes factor of 1 is considered no evidence, between 1 and 3 anecdotal, between 3 and 10 moderate, between 10 and 30 strong, between 30 and 100 very strong, and more than 100 extreme evidence that the data are in line with the alternative hypothesis. Conversely, a Bayes factor between 1/3 and 1 will be considered anecdotal; between 1/3 and 1/10, moderate evidence; between 1/10 and 1/30 strong evidence; between 1/30 and 1/100, very strong evidence; and less than 1/100, extremely strong evidence that the data are more likely under the null hypothesis (Wagenmakers et al., 2017).

#### Hypothesis 3

To examine if the difference between students in the low-alcohol group and high-alcohol group was more pronounced when AB was measured with the OOOT-adapt, we compared the confidence interval of the effect size comparing students who drink low vs. high amounts of alcohol derived from the OOOT-adapt with the confidence interval of the effect size derived from the OOOT.

## Results

### Descriptives

Of the 245 participants signing up for the study, four reported no alcohol consumption in the past month and were therefore excluded from the study. All four belonged to the low-drinking group. Of the remaining 241 participants, 157 identified themselves as drinking low amounts of alcohol (1–7 units a week), and 84 as drinking high amounts of alcohol (>14 units a week). However, there seemed to be anomalies in these self-identified groups and the quantity of alcohol use reported during the study (Table 1). We therefore decided to test our hypotheses based on the self-identified groups, as well as on groups based on the quantity of alcohol use reported during the study, that is, a group of low drinkers (1–10 units a week) and a group of high drinkers (11 or more units a week). In the following, we will refer to these two approaches as self-identified groups and reported groups. The numbers of participants per group and per condition for both approaches as well as the group characteristics are provided in Table 2.

**TABLE 1:** Use per week per group based on self-identification.

Quantity	Low (*n* = 157)	High (*n* = 84)
1–7	77	0
8–10	32	3
11–13	14	6
≥14	34	75

*Quantity, units of alcohol consumed in an average week in the past 30 days.*

**TABLE 2:** Group characteristics.

			Frequency		Quantity		State craving		Craving		Problems	
Group			Mean	SD	Mean	SD	Mean	SD	Mean	SD	Mean	SD
OOOT	Low	Self-identified (*n* = 96)	5.88	3.39	43.29	54.69	1.83	1.12	5.64	1.37	29.54	8.82
		Reported (*n* = 68)	4.93	2.84	22.94	10.50	1.71	1.05	5.37	1.27	26.93	6.64
	High	Self-identified (*n* = 31)	11.48	6.20	132.26	93.20	2.16	1.55	6.74	1.39	36.89	7.68
		Reported (*n* = 59)	9.93	5.33	113.49	89.43	2.15	1.40	6.53	1.41	36.38	8.94
OOOT- adapt	Low	Self-identified (*n* = 61)	6.82	3.95	41.25	30.80	1.79	1.10	5.69	1.38	29.24	7.52
		Reported (*n* = 44)	5.55	2.77	25.00	8.68	1.73	1.00	5.50	1.44	27.54	6.80
	High	Self-identified (*n* = 53)	12.04	5.44	117.66	57.23	2.94	1.63	7.13	1.96	37.69	9.08
		Reported (*n* = 70)	11.57	5.29	109.31	53.50	2.70	1.62	6.90	1.83	36.92	8.83

### Data Reduction of OOOT and OOOT-Adapt

Participants who fell more than three SDs below the mean accuracy were excluded. In the OOOT-adapt, five participants (two in the low- and three in the high-alcohol group) were excluded for this reason. These numbers were identical for the data reduction based on self-identified and reported alcohol use. Mean percentage of correct responses after exclusion per group is reported in Table 3. Trials with incorrect responses were deleted. Further, trials in which participants responded faster than 200 ms (i.e., expected anticipation errors) or fell more than three SDs below or above the mean response latency of that trial type were excluded. For the OOOT, no too slow or too fast responses were found. Also for the OOOT-adapt, there were no too slow responses, but there were too fast responses. In the self-identified low-alcohol group, one response was faster than 200 ms; in the self-identified high-alcohol group, six; in the reported low-alcohol group, none; and in the reported high-alcohol group, seven responses were faster than 200 ms and therefore deleted.

**TABLE 3:** Percentage correct per group.

		OOOT		OOOT-adapt	
Group		Mean	SD	Mean	SD
Low	Self-identified	69.05	22.61	86.04	8.36
	Reported	69.98	21.83	86.90	8.14
High	Self-identified	70.93	23.29	85.62	9.15
	Reported	68.69	23.81	85.19	9.02

### Hypothesis 1: Does the OOOT-Adapt Have Better Internal Consistency Than the Original Version of the OOOT?

Internal consistency calculated with the split-half method and the alternating method and the related confidence intervals are reported in Table 4. The Fisher *Z* test was used to statistically compare the internal consistency coefficients of the OOOT and the OOOT-adapt. Internal consistency as calculated via the split-half method showed that the internal consistency of the OOOT-adapt was indeed higher than that of the OOOT. This was not consistently the case for the internal consistency as measured with the alternating method.

**TABLE 4:** Internal consistency (Spearman–Brown).

			Low				High			
			OOOT	OOOT-adapt	*Z*	*P*	OOOT	OOOT-adapt	*Z*	*p*
Self-identified	Eng	Split-half	0.07 (−0.14; 0.28)	0.40 (0.16; 0.60)	2.113	0.017	−0.21 (−0.54; 0.17)	0.44 (0.18; 0.64)	2.904	0.002
		Alternating	0.19 (−0.02; 0.38)	0.24 (−0.02; 0.47)	0.313	0.377	0.48 (0.14; 0.72)	0.61 (0.40; 0.76)	0.788	0.215
	Dis	Split-half	−0.15 (−0.35; 0.06)	0.26 (0.00; 0.48)	2.494	0.006	0.19 (−0.19; 0.52)	0.66 (0.47; 0.79)	2.544	0.005
		Alternating	0.01 (−0.20; 0.22)	0.47 (0.24; 0.65)	2.989	0.001	0.53 (0.20; 0.75)	0.74 (0.58; 0.84)	1.866	0.031
Reported	Eng	Split-half	0.01 (−0.24; 0.26)	0.35 (0.05; 0.59)	1.782	0.037	−0.06 (−0.32; 0.20)	0.47 (0.26; 0.64)	3.149	0.001
		Alternating	0.16 (−0.09; 0.39)	0.22 (−0.09; 0.49)	0.312	0.377	0.48 (0.25; 0.66)	0.57 (0.38; 0.71)	0.688	0.246
	Dis	Split-half	−0.37 (−0.57; −0.14)	0.49 (0.22; 0.69)	4.635	<0.001	0.14 (−0.13; 0.39)	0.54 (0.34; 0.69)	2.558	0.005
		Alternating	0.19 (−0.06; 0.42)	0.53 (0.27; 0.72)	1.995	0.023	0.33 (0.08; 0.54)	0.66 (0.50; 0.78)	2.458	0.006

*Eng, engagement; dis, disengagement. Internal consistency 95% CI around Spearman–Brown coefficients are given in parentheses.*

### Hypothesis 2: Do Student Who Drink High Amounts of Alcohol Have a Stronger AB to Alcohol Cues Than Students Who Drink Low Amounts of Alcohol?

Mean AB scores and outcomes of the one-tailed independent *t* tests are reported in Table 5. Taking into account the adjusted α of 0.025, the results showed that only the OOOT-adapt was able to differentiate between the low- and high-alcohol group. Specifically, individuals in the high-alcohol group have more attentional engagement with alcohol cues than individuals in the low-alcohol group. This was the case when the groups were assigned based on self-identified alcohol use, as well as the reported amount of used alcohol. Bayes factors showed moderate to strong evidence that there are no differences between the groups on engagement and disengagement bias as measured with the OOOT or on the disengagement bias as measured with the OOOT-adapt.

**TABLE 5:** Mean attentional bias scores and one-tailed independent *t* tests.

	OOOT					
	Self-identified					
	Low (*n* = 88)	High (*n* = 29)	*t*	*p*	BF_10_	Cohen *d*
Engagement	−391.70 (470.60)	−373.75 (560.16)	−0.170	0.433	0.255	−0.036
Disengagement	728.75 (624.34)	819.08 (658.80)	−0.666	0.254	0.399	−0.143

	**Reported**					
	**Low (*n* = 63)**	**High (*n* = 57)**	***t***	***p***	**BF_10_**	**Cohen *d***

Engagement	−389.11 (470.66)	−507.46 (727.23)	1.068	0.856	0.102	0.195
Disengagement	688.41 (549.49)	934.39 (854.01)	−1.894	0.030	1.871	−0.346

	**OOOT-adapt**					

	**Self-identified**					
	**Low (*n* = 59)**	**High (*n* = 50)**	***t***	***p***	**BF_10_**	**Cohen *d***

Engagement	133.73 (227.35)	257.26 (313.77)	−2.377	0.010	2.463	−0.457
Disengagement	−241.36 (212.45)	−266.53 (350.95)	0.461	0.677	0.224	0.089

	**Reported**					
	**Low (*n* = 42)**	**High (*n* = 67)**	***t***	***p***	**BF_10_**	**Cohen *d***

Engagement	101.15 (199.92)	246.33 (301.82)	−3.014	0.003	5.693	−0.542
Disengagement	−227.19 (222.21)	−269.02 (316.24)	0.749	0.772	0.267	0.147

### Hypothesis 3: Is the Difference Between the Low- and the High-Alcohol Group More Pronounced When AB Was Measured With the OOOT-Adapt When Compared With the OOOT?

It was originally planned to compare the confidence intervals of the analyses of the OOOT and the OOOT-adapt. However, given the findings, this became redundant. That is, these analyses were planned on the premises that the tasks would provide relatively similar outcomes and group differences, yet one might be more pronounced than the other. However, the OOOT gives a negative attentional engagement score, and the OOOT-adapt, a positive attentional engagement score. For the attentional disengagement scores, this is reversed, but also here the tasks provide very different outcomes. Furthermore, only the OOOT-adapt showed a significant difference between the low- and high-alcohol group.

## Discussion

The current study showed that using more distinct non-alcohol contrast categories, adding practice trials and increasing the number of trials, having participants perform the AB assessment task in an alcohol-relevant context, and assessing AB in high-drinking individuals resulted in increased internal consistency of the alcohol AB measure. The updated version of the task, called the OOOT-adapt, was also able to differentiate between participants who drank low amounts of alcohol and those who drank a high amount of alcohol.

In accordance with our first hypothesis, we found the internal consistency of the AB indices to be higher when measured with the OOOT-adapt than when measured with the original OOOT. This was especially true when the internal consistency of the tasks was calculated using the split-half method. When calculating the internal consistency with the alternating method, the internal consistency of the OOOT-adapt was significantly higher for the disengagement bias compared with the OOOT. Although the same tendency was evident for the engagement bias, the difference between the OOOT-adapt and OOOT did not reach significance. Similar results in which the split-half method revealed higher internal consistency were found in a previous study (Jonker et al., 2019). One explanation for this apparently consistent difference between both ways of allocating trials to one or the other half could be that the split-half method is less sensitive to variable carryover effects of individual trials and reflects therefore a more stable reflection of the process of interest. In addition, the findings indicated that the internal consistency of the OOOT-adapt was most favorable in the group of participants who drank high amounts of alcohol. This is in line with the idea that AB measures are more stable in individuals where the salience/motivational relevance of the cues is higher (Field and Christiansen, 2012), generally individuals who drink more frequent and higher amounts. Based on the current findings, one can expect the reliability of the OOOT-adapt to be even better when assessing AB in a clinical sample. Therefore, the reliability of the OOOT-adapt might further be tested in future research including treatment-seeking individuals diagnosed with substance use disorder. Especially, as the current sample was restricted to a homogenous sample of university students, it seems important to test the generalizability of results in the clinical range.

In accordance with our second hypotheses, participants drinking high amounts of alcohol showed a stronger AB for alcohol cues than participants drinking low amounts of alcohol. This was only the case when assessing AB with the OOOT-adapt (making our third hypotheses redundant). That is, the OOOT-adapt successfully differentiated between low- and high-drinking individuals, and results showed that high-drinking individuals engage faster their attention with alcohol cues than low-drinking individuals. This difference was even more pronounced when the calculation was based on participants’ reported amount of used alcohol in the past month when compared with the self-identified average amount of used alcohol. With regard to the disengagement bias, there was no difference between low- and high-drinking individuals when measured with the OOOT-adapt, which seemed in contrast with the findings of the previous study in which alcohol use was related with disengagement bias but not engagement bias when measured with the OOOT (Heitmann et al., 2020). Looking more closely, a similar trend was evident in the current study, but remained non-significant (after the correction of α for multiple comparisons), and also the Bayes factor indicated no clear difference of disengagement bias between groups when AB was assessed with the original OOOT (i.e., when the calculation was based on the reported amount of consumed alcohol in the past month). In addition, taking the low internal consistency of the OOOT in the previous study, as well as in the current study into account, the meaning of this finding remains inconclusive. In contrast, the OOOT-adapt revealed itself as a promising task to be used as a measure of individual differences as it was able to differentiate between low- and high-drinking individuals and at the same time showed improved internal consistency (e.g., McNally, 2019). Future research might want to investigate the predictive validity of the AB indices as derived from the OOOT-adapt regarding alcohol use and craving.

### Strengths and Limitations

This study has several strengths, such as the high number of participants and the blinded allocation to one of the two conditions. There are also some limitations to bear in mind when interpreting the results of the current study. First, although the administration of AB using the OOOT-adapt in an alcohol-relevant context was a relevant adaptation, it also entails some disadvantages. That is, the current design of the study, in which the OOOT was administered in the laboratory and the OOOT-adapt in the bar, does not allow disentangling whether the adaptation to the task itself or the context lead to increased internal consistency of the OOOT-adapt. However, this approach allowed increasing the chance of a reliable and valid measure. Knowing that the adaptations indeed improved the reliability of the AB measure, a next step for future research could be to test to what extent the increased internal consistency can be attributed to the optimization of the task, or the context, or whether they have both summatively contributed to the improvement. It is also conceivable that the adaptation to the context might have reduced the reliability of the OOOT-adapt. That is, participants might have been more distracted in the bar context than when completing the task in the laboratory. Although the administration of the task took place in the afternoon when (almost) no visitors were present, we cannot rule out that participants were distracted at any point from the task. However, based on the percentage of correct responses, there is no indication that participants in the OOOT-adapt condition who completed the task in the bar made more mistakes. Second, the current study design does not allow disentangling which adaptations of the OOOT-adapt lead to improved internal consistency. For example, there is evidence that the number of trials from a task can influence its reliability (e.g., Tavakol and Dennick, 2011; Ataya et al., 2012). It might therefore be that the larger number of trials of the OOOT-adapt might partially explain its improvement regarding internal consistency. The number of trials from the OOOT-adapt was actually comparable with other AB measures (e.g., Townshend and Duka, 2007; Pennington et al., 2020). Nevertheless, future research might want to disentangle which adaptations of the OOOT-adapt are relevant regarding its reliability, for example, the influence of the number of trials and in particular the number of trials necessary to reliably measure the process of interest (i.e., AB). Third, although the OOOT-adapt showed improved internal consistency, it did not reach a value that is considered as a “good” reliability coefficient (≤0.8) based on commonly reported thresholds (Clark and Watson, 1995). This might relate to the fact that the task follows an unblocked task design in which trials are randomly presented, the use of divers images, and/or the fact that the task was assessed in a non-clinical sample (see above; Ataya et al., 2012). Furthermore, it has been argued that the commonly used thresholds as defined to assess reliability of questionnaires might not hold for measuring processes such as AB based on reaction times (e.g., Elgersma et al., 2019). Fourth, it might be important to consider that there was a difference in the number of alcohol stimuli on the screen between the alcohol distractors trials that are critical to compute the disengagement bias and the alcohol target trials that are critical to calculate engagement bias (19 vs. 1). The presentation of multiple alcohol images in the alcohol distractors trials was necessary to ensure that the initial attention would be typically directed on an alcohol image, thereby allowing to test how much difficulty participants would experience to redirect their attention to find the single neutral odd-one-out stimulus. However, this difference in the number of alcohol images on the screen between both types of trials might have differentially affected participants’ response times, for example, by eliciting stronger craving or distraction from the task when responding to alcohol distractors trials showing multiple alcohol images. In addition, one could also speculate that the multitude of alcohol images elicited multiple instances of engagement next to a difficulty to disengage. Future research might want to investigate to what extent slowed responding to alcohol distractors trials indeed reflects disengagement bias, for example, by using eye-tracking during task performance. Fifth, there were discrepancies between individuals’ self-identified average amount of used alcohol and what was later reported during the study about the past month. As indicated, we therefore completed all analyses based on self-identification prior to the study and based on the reported amount of consumed alcohol. Generally, results seem to point in the same direction, and we therefore do not expect that group allocation influenced the results in a relevant way.

### Conclusion

Adapting the original OOOT by using more distinct contrast stimulus categories and adding practice trials and more relevant trials, as well as assessing this task in an alcohol-relevant context and in high-drinking individuals, indeed improved the internal consistency of the AB measure. This improved task also showed superior criterion validity as the engagement bias index of the OOOT-adapt could differentiate between low- and high-drinking individuals, thereby adding to the evidence that AB might be involved in substance use. To further test the utility of the OOOT-adapt to index AB, a critical next step would be to evaluate whether the promising psychometric properties also hold in the clinical range, and whether the AB measure not only remains consistent within one assessment procedure but also shows stability over time (test–retest reliability). If proven to be a reliable measure, the OOOT-adapt can enhance the field of research by serving as a task to further test the causal role of AB in addiction.

## Data Availability Statement

The raw data supporting the conclusions of this article will be made available by the authors, without undue reservation.

## Ethics Statement

The studies involving human participants were reviewed and approved by Ethical Committee of Psychology (ECP) of the University of Groningen. The patients/participants provided their written informed consent to participate in this study.

## Author Contributions

JH and NJ were responsible for the data collection. NJ conducted the analysis. JH drafted this manuscript. All authors contributed to the design of the study and further contributed to the writing process and approved this final manuscript.

## Conflict of Interest

The authors declare that the research was conducted in the absence of any commercial or financial relationships that could be construed as a potential conflict of interest.

## Footnotes

1 https://osf.io/yfm25
